# The effects of COVID-19 on New York State’s Drug User Health Hubs and syringe service programs: a qualitative study

**DOI:** 10.1186/s12954-023-00742-9

**Published:** 2023-02-02

**Authors:** Mercy Ude, Czarina N. Behrends, Shea Kelly, Bruce R. Schackman, Allan Clear, Rebecca Goldberg, Kitty Gelberg, Shashi N. Kapadia

**Affiliations:** 1 Weill Cornell Medical College, New York, NY 10065 USA; 2 Department of Population Health Sciences, Weill Cornell Medical College, New York, NY 10065 USA; 3 New York State Department of Health, New York, NY 10007 USA; 4 Division of Infectious Diseases, Weill Cornell Medical College, New York, NY 10065 USA

**Keywords:** People who inject drugs, Harm reduction, Syringe services program, Telemedicine, COVID-19

## Abstract

**Background:**

Syringe service programs (SSPs) deliver critical harm reduction services to people who inject drugs (PWID). Some SSPs in New York State received enhanced funding to provide additional services to combat opioid overdose fatalities. These SSPs, known as Drug User Health Hubs, provide buprenorphine for the treatment of opioid use disorder and other health-related services in addition to their syringe services. While the COVID-19 pandemic posed widespread challenges to the delivery of health services nationwide, the effect of the pandemic on SSPs uniquely impacts PWID. This study examines the impact of COVID-19 on service delivery of Drug User Health Hubs and stand-alone SSPs in New York State.

**Methods:**

Between July 2020 and September 2020, we performed eleven semi-structured virtual interviews with staff from three Health Hub SSPs and three stand-alone SSPs. The interviews explored the effect of the COVID-19 pandemic on SSPs and their clients as well as the changes implemented in response. Interviews were recorded and transcribed. We performed content analysis to identify emerging themes from the data.

**Results:**

Due to the COVID-19 pandemic, some SSPs temporarily shut down while others limited their hours of operation. SSPs modified their service delivery to maintain syringe services and naloxone distribution over other services such as STI and HCV testing. They virtualized components of their services, including telemedicine for the provision of buprenorphine. While SSPs found virtualization to be important for maintaining their services, it negatively impacted the intimate nature of client interactions. Participants also described the impact of the pandemic on the well-being of PWID, including isolation, worsened mental health challenges, and increased drug overdoses.

**Conclusions:**

In response to the COVID-19 pandemic, SSPs demonstrated innovation, adaptability, and togetherness. Despite the challenges posed by the pandemic, SSPs continued to be key players in maintaining access to sterile supplies, buprenorphine, and other services for PWID. In addition to adapting to COVID-19 restrictions, they also responded to the dynamic needs of their clients. Sustainable funding and recognition of the critical role of SSPs in supporting PWID can help to improve outcomes for PWID.

## Introduction

Syringe Service Programs (SSPs) play a crucial role in delivering critical services to people who inject drugs (PWID). While diverse in their structures and delivery models, SSPs are community-based programs grounded in harm reduction principles and community advocacy. In addition to syringe services, SSPs typically offer additional services for PWID including opioid overdose education, naloxone distribution, HIV and hepatitis C (HCV) testing, and linkage to medical care [1, 2]. There is robust evidence supporting the effectiveness of SSPs in the prevention of HIV and HCV among PWID [3–10].

The COVID-19 pandemic has had a significant impact on health delivery nationwide. New York was an early epicenter of the COVID-19 pandemic. In March of 2020, New York State declared a state of emergency due to the rapidly increasing incidence of COVID-19, followed by an executive order mandating the closure of all non-essential businesses and offices [11]. With 203,000 confirmed cases by May of 2020, New York State accounted for over half of all confirmed COVID-19 cases in the US at that time [12]. As the Department of Health and other state agencies were redirected toward the COVID-19 response, the state struggled to maintain consistent funding for SSPs and other services. Data from New York shows that the pandemic led to increased mental health challenges for PWID, with reduced access to SSPs and medication for opioid use disorder (MOUD) [13]. During the pandemic, some SSPs shut down their sites while others had reduced availability of services [14]. Under the pressures of COVID-19, SSPs made changes to their delivery approach to maintain the safety of staff and clients, and prioritized syringe and naloxone distribution over other services [15].

In New York State, some SSPs were funded to implement additional programming targeted toward reducing opioid overdose fatalities. These SSPs, called Drug User Health Hubs (“Health Hubs”), provide typical harm reduction services, and also provide on-site buprenorphine for MOUD. Few SSPs offer on-site buprenorphine in the United States [16], therefore, Health Hubs provide an opportunity to learn about this model of offering buprenorphine in an accessible, trusted environment. During the COVID-19 pandemic, Health Hubs and other SSPs that provide medical services faced additional challenges from COVID-19 not yet described and developed new innovations to overcome these challenges.

Given the critical role that SSPs play in their communities, COVID-mediated changes in service delivery at SSPs had the capacity to worsen access to care and health outcomes for PWID, but also to mitigate the harm being caused by the pandemic. As a part of an ongoing evaluation of the Drug User Health Hub program, this study aims to understand how the COVID-19 pandemic affected service delivery at Health Hubs and other SSPs in New York State in 2020.

## Methods

### Research team

The methods of this study are reported according to the Consolidated Criteria for Reporting Qualitative Research (COREQ) checklist [17]. The research team consisted of PhD and MPH researchers and clinician investigators at two academic institutions and the New York State Department of Health. Interviews were conducted by a study coordinator at the Department of Health (SK) and either of two University Investigators (SNK or CNB). Qualitative analysis was conducted by University Investigators (MU, SNK). University investigators did not have pre-existing relationships with study subjects, while Department of Health staff (SK) was familiar with subjects through implementation of the Health Hubs program.

### Parent evaluation design

This qualitative study is embedded in a larger evaluation study of the Health Hubs program. In that study, Health Hubs implementation is being examined and outcomes are being compared between Health Hubs and “stand-alone” SSPs. Both Health Hubs and stand-alone SSPs are located in New York State and provide essential syringe services and other harm reduction services. Health Hubs provide additional services including buprenorphine treatment.

### Sample

Participants included key management staff from 3 Health Hubs and 3 stand-alone SSPs in New York State. All of the programs participating in the study are part of larger multi-site programs with one site from each program participating in the study, except for program 3, which had two separate sites participating (Table 1). One to two staff members from each program were interviewed. A total of 6 staff members from 3 Health Hubs and 5 staff members from 3 stand-alone SSPs were included in the study. Participants held management or administrative positions within their programs, such as program directors, supervisors, and coordinators. To maintain anonymity, titles for individual participants are not reported.

**Table 1:** Program description

Program	Site	Number of interviews	Program type	Number of unique clients in calendar year 2020
1	A	2	Stand-alone SSP	< 500
2	B	2	Stand-alone SSP	< 500
3	C	1	Stand-alone SSP	500–1000
	D	2	Health Hub SSP	> 2000
4	E	2	Health Hub SSP	500–1000
5	F	2	Health Hub SSP	500–1000

### Data collection

We conducted eleven semi-structured interviews of staff from Health Hubs and stand-alone SSPs. Interviews were conducted over WebEx video-conference software between July and September 2020 and ranged from 80 to 110 min in length. Interviews were recorded and transcribed. Each interview consisted of two interviewers and one respondent. Interview guides examined multiple domains related to Health Hub and syringe services program implementation. Analyses in this manuscript focus on responses about the COVID-19 pandemic and (1) staffing and capacity of programs (2) service offerings and changes, (3) strategies and adaptations to maintain service delivery, (4) well-being of program clients. Participants were not compensated for the interviews.

### Data analysis

Interviews were coded using content analysis after data collection was completed [18]. Two university researchers (MU, SNK) coded each transcript independently using the NVivo software [19]. Initial codes were developed deductively from previous literature on the topic. These codes included: *COVID-19, Telemedicine, Buprenorphine Access, HIV/HCV testing, Outreach, and other themes from prior studies.* Additional codes were added inductively as they emerged from the data. New codes that emerged from this data included: *isolation, drug supply, overdose, staff mental health, remote work, PPE and Social Distancing, and others.* Researchers discussed themes that emerged across categories and converged related codes into themes, then refined them through continued discussion.

The results encompass findings from both Health Hub SSPs and stand-alone SSPs unless specified.

## Results

Three major domains emerged from the data, each containing 3–5 separate themes: (1) operational changes resulting from the pandemic, (2) changes to the individual services delivered, and (3) the effect of the pandemic on the well-being of SSP clients.

### Operational changes resulting from the pandemic

#### Shutdowns and reduced hours

During the COVID-19 pandemic in 2020, some SSPs temporarily closed while others reduced their hours of operation as directed by state guidelines. However, one program that had been operating under a limited schedule emphasized the desire to continue to perform essential tasks: “Even if the governor would’ve not let us come in…we were starting to get busier so we were actually gonna start reviewing and seeing if we can just go back to our normal schedule. (A)” Due to the pandemic’s effect on funding, one program described furloughing a number of its staff during the pandemic and the significant impact this had on their ability to maintain services:

Because we had kind of a halt in reimbursement from New York State, we did have to furlough several staff… We just weren't getting money in enough to be able to continue to pay people. So we went a month. We furloughed people starting July 4, and July 6 that faucet turned and things kind of flowed back into the agency… But that month, that 30 days, was tough. It was figuring out what we needed to do and really just maintaining the programs as best we could with the staff. (B)

Designation as an ‘Essential Service’ became an important lifeline for SSPs, allowing programs to keep staff onboard and continue to service their target population.

#### Confusion and uncertainty

During the early pandemic, SSPs rapidly made operational changes to their programs in an effort to keep clients and staff safe and comply with government guidelines. They described periods of uncertainty, during which they were unsure of which types of operational changes would need to take place and for how long these changes would last: “You know it's been tough on staff, because there's so many changes just because no one knows. You know the distance guidelines comes out every day and we have to change things up, but they've [the staff] been so adaptable and you know almost like kind of brought us together more. (F)”.

This period was also characterized by confusion about how to fulfill both minor and major tasks given the rapidly evolving environment. For one program, common tasks like placing referrals became more complicated with the new work environment: “I know like our department of human services and stuff, it was like, the door was locked and the way people had to access it were in ways that just weren't realistic. Oh, you can scan this paperwork. And you're like, who has a scanner? I don't know. Maybe my kid has a scanner or something. I don't necessarily think I have a scanner. (C)” Another program described their attempts to cope with the changing environment: “You know, the way we're operating right now, it's literally 1 week at a time of how we're doing things, how things are changing, what the agency has decided is a priority versus what the agency has decided to no longer be a priority. (F)”.

SSPs were unable to prepare their clients for changes in access due to rapidly changing restrictions. Whereas prior closures would have been communicated to clients and to the community beforehand, the uncertainty from the COVID-19 pandemic made it difficult for programs to appropriately alert their clients of shutdowns or closures.:

When we shut down, it was literally from one day to the next. We were in the office one day. The next day, nobody goes to the office… That was hard for me to swallow because typically, if we're going to close for the holidays… we give a well-advanced notice to our participants. We make sure they have enough supplies. We make sure that, you know, we link them to any services that they may need or they think they may need while we're closed. We weren't able to do that. We weren't able to give our participants any notices. (B)

#### Limited access to personal protective equipment

In response to the COVID-19 pandemic, programs implemented social distancing, symptom screening, and personal protective equipment (PPE) policies. However, SSPs found it to be “a huge battle trying to get PPE (B)” during the early pandemic for both staff and clients. For some programs, this was a significant barrier to maintaining full operations or resumption of services. SSPs overcame this barrier through support from the community and other partners. Several programs reported receiving masks, other PPE, or hand sanitizers as donations from businesses in the community or local agencies. One program describes having insufficient access to hand sanitizer but received enough donations of PPE to distribute these supplies to clients: “We unfortunately do not have access to hand sanitizer to give them. Because these are hot commodities these days. But we've been able to get donations from a lotta local agencies and we provide reusable masks to all of our clients who need them. (A)”.

#### Changes to physical spaces

In order to adhere to social distancing guidelines during the pandemic, SSPs also made changes to their physical spaces. Such changes included providing services outdoors where possible, performing transactions through windows, reorganizing office spaces, and closing down spaces where clients typically gather such as lobbies and lounges.

These changes affected the intimate nature of their interactions with clients typical of SSPs. Staff described the physical environment after COVID changes as less personal and more clinical and transactional, contradicting the normally casual and welcoming environment that characterizes SSPs: “It's a lot less homey and a lot more clinical than we would like, but we understand right now, that's just how it has to be, that we don't want to have a lot of things out that we have to sanitize and we have to keep things moving, that we can't have benches out for people to hang out on and have the TV on and stuff. (C)”.

### Changes to individual services delivered

#### Telemedicine provision of buprenorphine

All the Health Hubs included in this study provided buprenorphine in addition to their services as SSPs. During the pandemic, Health Hubs were able to continue providing buprenorphine though rapid implementation of telemedicine. Programs utilized phone calls and video conferencing software to provide buprenorphine: “Our nurse practitioners in the support staff that help with the low-threshold buprenorphine stuff from a hub program standpoint, were really I think excited to be able to do appointments by telephone, and like video calling type of things at first March, April in the early part of the pandemic. (D)”.

The SSPs generally found these telemedicine programs to be successful: “I mean, it was really great during, like, the peak of our crisis… Because it offered the opportunity for our provider to still connect with our patients, and it didn't require patients to come in, it included the opportunity to engage or do inductions with new patients, and it was really seamless and easy. (E)” Some Health Hubs provided phones on-site to ensure that patients could engage in telemedicine regardless of personal phone access. Program staff described telehealth as a platform that they would like to continue after the end of the pandemic.

While SSP staff found telemedicine to be seamless on their end, they felt that provider-patient interactions were not as strong as they had been in-person.

We’ve only had the low-threshold buprenorphine stuff going for a few years here, but it seems like part of what makes that program work is that the rapport and the trust of the connection between that staff and the people that are taking part in the program. And it just was cool to still be able to prescribe the medication and make some connection with them, but telehealth doesn’t capture the interaction as well as in-person one does, so that’s been a struggle. (D)

#### HIV/STI testing was deprioritized

The pandemic required programs to rapidly adjust their service delivery given the new COVID prevention policies and the reduced capacities of many SSPs. SSPs halted their HIV and HCV testing, while developing innovative strategies to ensure that buprenorphine, syringe services, and naloxone distribution continued. One explanation for the de-prioritization of HIV testing and other rapid testing was that these services were too difficult to perform within the limitations of COVID-19 social distancing policies. Additionally, HIV and STI testing were not perceived to be as urgent as their ‘core services’ which include syringe services and naloxone distribution. For one SSP, they considered how counseling patients after a positive rapid test would be difficult given the constraints of COVID.

But for a while we weren't doing the rapid testing… It was more like we slipped more into doing the syringes, Narcan. So, that kind of affected it just because we were trying to make sure that we were socially distant, for their sake and for our sakes. And we were also trying to be mindful about how long we would spend with a patient as well. So, we didn't want to sit down and do like an intervention that was going to take a half-hour or something. So, that was a piece. We really pulled into some of the very, very core services for a while. (C)

While most centers halted HIV/STI testing, one program implemented a delivery system to address the challenges of infectious disease testing during the pandemic.

Pretty much we were doing all services remotely. Even with HIV testing, we were actually mailing out the home HIV test kits so that people who felt they were at risk could still test themselves. We would do a pretest screening where we would talk about, "What are you concerned about? What are some of the reasons? Do you have concerns and that's why you're asking for one?" (B).

#### Syringe distribution delivery was adapted to address storefront closures and increased need

Due to the pandemic, some SSPs pulled back their mobile vans and closed down storefronts. In order to maintain syringe distribution, they developed alternative systems such as collaboration with local pharmacies, delivery systems, and special arrangements. One center developed a new text message system to facilitate syringe delivery to their clients. While syringe deliveries were a novel COVID innovation for some programs, SSPs considered continuing the system given its efficiency and wider reach: “More specifically in terms of syringe exchanges, we are leaning toward, ‘Does it make sense to keep the mobile units when we built this sort of really efficient system of doing deliveries, and then folks may be on the peer delivery syringe model’—which allows us, really, to reach more people. (E)”.

In order to make transactions safer for clients and staff, one program described pre-packaging their supplies to minimize COVID exposure: “We've pre-packaged more things than we used to. I'm not quite sure if we're going to hold on to that or not, but we thought that because of COVID, that the faster we could do the [syringe] exchange the better. (C)” This SSP also packaged other harm reduction supplies and PPE, such as cookers, cotton, and gloves, in order to streamline their interactions with clients.

During the pandemic, SSPs were providing more syringes to clients than before the pandemic. They attributed this to an increased demand in syringe services stemming from the anticipation of future closures or reduced availability of syringe services. Programs also believed there to be more secondary exchange of sterile supplies occurring during the pandemic, with some clients not presenting to SSPs due to barriers of the pandemic. SSPs preemptively increased their supply of syringes and other harm reduction supplies (fentanyl strips, Naloxone kits) given their own uncertainty of what operational changes could lie ahead: “We started loading clients up with 300 syringes at a time—especially early-on when we anticipated that we could close entirely. And we weren't going to have access to anything. So we gave out a few Narcan kits, those types of things, extra Fentanyl test strips. (E)”.

#### New roles in the midst of COVID

As the needs of PWID evolved during the pandemic, SSPs adapted to meet them. One way that SSPs helped to meet emerging needs was by becoming a source of COVID-related information for their clients. Programs believed that their clients tended to be more vulnerable, having less access to digital information-sharing platforms such as social media and television news. Therefore, some clients first learned of the pandemic via their interactions with SSP staff. Programs soon took on the role of providing COVID information, including harm reduction related information, as well as dispelling misinformation about the pandemic.

A lot of our clients didn't know. They had no idea what was going on. Because if you don't have access to social media, if you're not kind of running in a circle where people are talking about this, how would you know? And it was really upsetting to know how many people weren't informed. So while we don't provide COVID-specific services, we have been, since that time, providing information to our clients about COVID and about these hygiene practices that can help folks. (A)

Additionally, as COVID worsened pre-existing barriers for their target population such as food and shelter, SSPs helped to meet these needs by providing grocery vouchers, shelter referrals, hygiene kits and PPE. One center described providing gift cards in addition to grocery vouchers: “And then because of COVID, we've been able to give folks our food vouchers, those gift cards… We're able to give people that. And our clients are really thankful for that. Because food stamps don't cover hygiene products, right?… Every client gets one per month… And how thankful they've been. Because they've never had that service on the van before (A).”

#### Virtualization

In addition to providing buprenorphine via telemedicine, SSPs made efforts to virtualize other aspects of their service delivery as well. Programs provided virtual Narcan trainings, virtual harm reduction counseling, and held other virtual groups. Virtualization allowed SSPs to maintain their outreach programs, community education, and social check-ins for both staff and clients.

So very quickly we were able to set up Zoom meetings, Zoom support groups that people could come to, even one-on-one. So to be able to really continue with the service delivery was something that was really important not only to the administration here, but also to our staff. They didn't want to leave their clients isolated and alone 'cause they knew that things could get much worse if that was the case. They really wanted to make sure that those services continued in whatever way we could. (B)

SSPs found virtual platforms to be an effective way of delivering services, with many SSPs intending to maintain a virtual option for their clients.

Barriers for some patients included poor access to Wi-Fi or smart devices, and technology illiteracy: “We are doing our opioid overdose prevention community training via Zoom. Our nutrition health education program, a lot of those clients are older, and so they were having trouble with logging onto Zoom and that type of thing (B).” Additionally, while virtual platforms were critical for maintaining service delivery for SSPs, they could not provide the level of intimacy and connection that in-person interactions provided. One program worried that with telehealth and other virtual models, they were not maintaining “enough of a connection with the population. (D)” Another SSP described how ‘zoom fatigue’ impacted their care delivery:

After a while, our patients started to ask about when they could come in. They wanted to see our provider… I've heard that trend…particularly around, like, recovery groups and peer recovery, like, everybody's zoomed out. Like, you know, there's only so many, like, Zoom meetings that you can go to; people miss, like, that face-to-face connectivity and – and we've started to see that. (E)

### Effect of the pandemic on PWID well-being

#### SSP drop-in space closures

While changes such as virtualization and closure of waiting rooms and gathering spaces were necessitated by the COVID pandemic, these changes meant less human connection and less time spent with clients. The population serviced by SSPs is particularly sensitive to the removal of safe spaces, given the stigma they experience within the larger society. For many clients, the closure of SSPs meant fewer safe spaces for PWID and more social isolation. SSPs felt that the closing of their physical spaces negatively impacted the mental well-being of their clients:

And I'm watching the repercussions of us closing due to COVID on TV. And, you know, that was very difficult for me to watch. And, I mean, I can imagine it was even more difficult for the participants… For some people, their only outlet for them – their only human connection for them is to come down to the exchange... Some people will come down, and they'll spend their half-hour, hour – in some cases, two hours – just wanting to talk; just wanting to connect with another human being. And since we built that trust and that relationship with most of our participants, you know, they know that they always have that...to closing down and then pretty much taking that away from them really impacted them. It really impacted them. (B).

#### Mental health challenges

During the COVID pandemic, social isolation, anxiety, and loss of social assistance negatively impacted the mental health of SSP clients. Programs felt that their client population was particularly affected by the social isolation that many people experienced during the pandemic. One staff member recounted how during the pandemic, “many individuals from recovered communities or substance use community became isolated. (E)” Another program described how this social isolation was compounded by the loss that PWID experienced during the pandemic: “And, you know, it made us a little sad. It did. It made me personally sad because when I saw the—I saw what was missing, and I saw that loneliness and that…and I saw, you know—and we heard—a lot of reports of their friends, their sisters, their mothers, their fathers overdosing and dying during that period. You know? (A)”.

SSPs described the stress and anxiety that their clients experienced during the pandemic. This stemmed from worsened social conditions and loss of social assistance, such as the closing of food pantries and difficulty accessing housing referrals: “When everything started closing down I think we started seeing—I mean, I think it was emotionally hurting our staff to see this, where they started closing food pantries, soup kitchens. And it was like everybody's like, ‘Well, COVID is happening so I'll close,’ but not really noticing how much that would impact so many of our clients, our community (A)”.

#### Increased drug overdoses

Programs worried about the increased overdoses in their communities during the COVID-19 pandemic. The rise in overdoses came at a time of limited access to clean syringes and supplies and worsened social conditions. Programs learned of these overdoses through anecdote from their clients, publicly shared information, and data-sharing collaborations with local stakeholders. These collaborations include taskforces for overdose prevention, police departments, emergency departments, county health departments, housing programs, and other community stakeholders.

The pandemic may have shifted the drug supply such that clients were accessing different drugs from different and unfamiliar suppliers. One proposed reason for increased overdoses in the community was fentanyl in the stimulant drug supply:

And there were situations where like I’ll remember one in late May… Three individuals died in a house, and what was obtained at the scene, you know, was a mixture of cocaine and fentanyl. And that matched some of these anecdotes that we had heard that people whose primary drug of choice was cocaine were falling out. Falling out in the way that one would from fentanyl. And of course those are people that didn’t tend to have like lots of Narcan on hand, didn’t tend to be people who had fentanyl test strips. (D)

Another proposed reason for more overdoses was an increase in substance use during the pandemic as a result of increased psychological stressors: “We've seen a lot more of that, like, this hopelessness, that I think drives some people's risky behavior. (E)” Finally, increased rates of solitary drug use, as a result of COVID-mediated social isolation, was also identified as a potential factor in increased overdoses in the community:

A lot of people have said of course the isolation – a big thing that we talk about is: never use alone. But when you're in a situation, you have to use alone. You're using alone. What happens then? … There've been so many talks about what it is. There're some people that just feel that isolation, that depression, and that increase, that mental health piece, and there's been an increase [in overdoses] because of that. (A)

## Discussion

This study investigated the impact of the COVID-19 pandemic on stand-alone SSPs and Drug User Health Hub SSPs during the summer of 2020 in New York State. The response of these SSPs to the COVID-19 pandemic was characterized by innovation, adaptability, and togetherness. SSPs adjusted all aspects of their programming including the spatial organization, the syringe service delivery model, and the treatment model for buprenorphine. They modified their systems to maintain the services they deemed feasible and most important, while minimizing COVID exposure to their clients and staff. They also took on new roles and provided additional services, such as providing COVID information and PPE, as the needs of their clients changed. Despite the emotional toll that the COVID-19 pandemic had on staff members, and structural challenges they experienced with funding and staffing, these SSPs demonstrated resilience as most recovered and reopened services to maintain service delivery.

Throughout the pandemic, SSPs maintained a sense of togetherness, a value that has been principal to the harm reduction community since prior to the COVID-19 pandemic. SSPs, which tend to operate on a small and more local scale, worked with other community stakeholders to share information regarding how COVID was impacting their community. They also leaned on their community for donations of PPE and other resources. Likewise, their clients looked to SSPs for COVID-related information and distribution of PPE, in addition to their SSP services. Importantly, they continued to prioritize engagement with their clients, via actions such as scheduling virtual check-ins and calls with clients.

While Drug User Health Hub SSPs offer a more comprehensive set of prevention and clinical services compared to the typical SSP, their primary services remain rooted in syringe and naloxone distribution. Despite the medical aspect of Health Hub service capabilities, both Health Hub SSPs and stand-alone SSPs prioritized syringe services and naloxone over services such as HIV and STI testing that could not be easily offered via telehealth during COVID-19. These findings are in line with other qualitative findings from SSPs in the United States [14, 15, 20]. Additionally, during the COVID-19 pandemic there was a national decrease in STI screening [21–23] and an increase in incidence of STIs [22, 24]. The use of at-home HIV tests by a program in this study provides one pathway for encouraging HIV testing among PWID during times of prolonged service disruption at SSPs.

SSPs were automatically designated as an “Essential Service’ in the state of New York [25], allowing those that closed their doors to reopen. Still, these programs needed to operate under PPE and social distancing policies, and also wanted to reduce COVID exposure for both their clients and their staff. In order to maintain their syringe services while reducing exposure to COVID, SSPs developed new approaches such as pre-packaging syringes and supplies and creating text message-based syringe delivery systems. SSPs also preemptively increased the quantity of syringes and naloxone distributed to clients during this time, consistent with findings from national quantitative data [2]. SSPs increased syringe distribution due to anticipation of (1) further closures or reduced capacity, (2) clients presenting to SSPs less frequently to minimize COVID exposure, and (3) increased secondary exchange of supplies. These innovations and changes in delivery models that were developed as a response to COVID-19 may persist even after restrictions related to the pandemic have been lifted.

The Health Hubs that provided buprenorphine were able to implement telemedicine, which has been noted elsewhere as a potential tool for improving access to buprenorphine initiation during the COVID-19 pandemic [26]. National data has shown that SSPs with MOUD on-site increased their provision of buprenorphine in 2020, with a modest increase in telemedicine-based buprenorphine [2]. On one hand, studies demonstrate that telehealth can reduce costs [27–30], maintain high patient satisfaction levels [31], and improve access to care [32]. However, during the height of the pandemic, a period during which telemedicine was often the sole option offered by programs for MOUD access, virtualization may have presented a new barrier to accessing care. For those without consistent access to internet or phones and for those with technological illiteracy, virtualization may have negatively impacted their ability to access services compared to the pre-pandemic period.

A unique finding of this study was how virtualization affected interactions between SSPs and their clients. SSPs commented that virtualization eroded the intimate nature of interactions between SSP staff and their clients during the pandemic. Given the negative health experiences of PWID with healthcare providers [33, 34], rapport and trusting relationships with providers and staff at SSPs are critical to effectively delivering harm reduction services and buprenorphine. In addition to changes in service delivery, virtualization impacted staff’s ability to provide emotional support to their clients and maintain strong engagement within PWID in their communities. While virtualization helped to mitigate disruptions in service delivery to PWID, it could not replicate the intimacy of in-person interactions. As in-person restrictions related to COVID-19 are no longer in effect in most areas, it remains to be seen how harm reduction programs utilize virtual care in their models. Hybrid models that combine virtual engagement with traditional in-person activities may be able to both increase access overall while maintaining the structures that build community and rapport with harm reduction providers.

Recent data from the Centers for Disease Control and Prevention (CDC) indicate a devastating increase in drug overdose deaths during the April 2020 to April 2021 period compared to the corresponding prior 12-month period [35]. Findings from our study suggest that SSP staff had been aware of the overdoses occurring in their communities. Our participants were particularly worried about a paucity of sterile syringes during this time due to disruption of SSP services. According to SSPs in New York State, potential causes for increased overdoses include disruptions in the drug market, more fentanyl in the drug supply, increased drug use as a result of psychosocial stressors, and riskier drug use behaviors such as more solitary drug use. Various studies have analyzed the relationship between the pandemic and overdose risk among PWID [36–39]. However, few of these studies have utilized SSPs, which are grounded in the drug user community, as a resource in understanding PWID and the factors that impact their experiences.

This study has several limitations. One limitation is that the findings are based on a selected sample of SSPs in a state that was severely affected by COVID-19 early in the pandemic. In particular, our sample includes Drug User Health Hub SSPs which have augmented funding to provide additional services. This is advantageous because these programs tended to have a broader spectrum of activity, including providing buprenorphine, which enriches the existing literature. However, some findings may not represent those of other SSPs. Additionally, this study does not include the direct perspectives of PWID, as client interviews were not included in the larger evaluation study that this project is embedded within. Another limitation is the cross-sectional nature of data collection. As the pandemic progressed and we learned more about COVID-19, SSPs likely continued to evolve. Our findings apply to the earliest months of the pandemic, and as policy responses adapted to changes in COVID incidence and severity, a number of changes to SSP operations that we observed may have reverted to their previous practice. However, this early data is helpful for informing a suitable emergency preparedness approach for SSPs and PWID in future.

This study adds to the literature describing the impact of COVID-19 and related restrictions on SSPs, while uniquely identifying themes that are relevant to SSPs providing an expanded array of services, including buprenorphine. During the height of the pandemic in New York State, SSPs were key players in maintaining access to sterile injection supplies and helped to maintain access to MOUD. Disruptions in their services have the potential to significantly worsen outcomes for PWID. With the persistence of the COVID-19 pandemic for 3 years, providing sustainable funding for SSPs and recognition of SSPs as critical and ‘essential’ community partners in ending the opioid epidemic can improve outcomes for PWID.
